# A novel expression system of domain I of human beta2 glycoprotein I in *Escherichia coli*

**DOI:** 10.1186/1472-6750-6-8

**Published:** 2006-02-10

**Authors:** Yiannis Ioannou, Ian Giles, Anastasia Lambrianides, Chris Richardson, Laurence H Pearl, David S Latchman, David A Isenberg, Anisur Rahman

**Affiliations:** 1 Centre for Rheumatology, Department of Medicine, University College London, Arthur Stanley House, 40-50 Tottenham Street, London. W1T 4NJ. UK; 2 Molecular Medicine Biology Unit, Institute of Child Health, University College London, 30 Guilford Street, London. WC1N 1EH. UK; 3 Cancer Research Campaign Centre for Cell and Molecular Biology and Section of Structural Biology, Institute of Cancer Research, 123 Old Brompton Road, London, SW7 3RP. UK

## Abstract

**Background:**

The antiphospholipid syndrome (APS), characterised by recurrent miscarriage and thrombosis, is a significant cause of morbidity and mortality. Domain I (DI) of human beta 2 glycoprotein I (β_2_GPI) is thought to contain crucial antibody binding epitopes for antiphospholipid antibodies (aPL), which are critical to the pathogenesis of APS. Expressing this protein in bacteria could facilitate studies investigating how this molecule interacts with aPL.

**Methods:**

Using a computer programme called Juniper, sequentially overlapping primers were designed to be used in a recursive polymerase chain reaction (PCR) to produce a synthetic DI gene. Specifically Juniper incorporates 'major' codons preferred by bacteria altering 41 codons out of 61. This was cloned into the expression plasmid pET(26b) and expressed in BL21(DE3) *Escherichia coli *(*E. coli*). By virtue of a *pel*B leader sequence, periplasmic localisation of DI aided disulphide bond formation and toxicity was addressed by tightly regulating expression through the high stringency T7*lac *promoter.

**Results:**

Purified, soluble his-tagged DI in yields of 750 μg/L bacterial culture was obtained and confirmed on Western blot. Expression using the native human cDNA sequence of DI in the same construct under identical conditions yielded significantly less DI compared to the recombinant optimised sequence. This constitutes the first description of prokaryotic expression of soluble DI of β_2_GPI. Binding to murine monoclonal antibodies that recognise conformationally restricted epitopes on the surface of DI and pathogenic human monoclonal IgG aPL was confirmed by direct and indirect immunoassay. Recombinant DI also bound a series of 21 polyclonal IgG samples derived from patients with APS.

**Conclusion:**

By producing a synthetic gene globally optimised for expression in *E. coli*, tightly regulating expression and utilising periplasmic product translocation, efficient, soluble *E. coli *expression of the eukaryotic protein DI of β_2_GPI is possible. This novel platform of expression utilising pan-gene prokaryote codon optimisation for DI production will aid future antigenic studies. Furthermore if DI or peptide derivatives of DI are eventually used in the therapeutic setting either as toleragen or as a competitive inhibitor of pathogenic aPL, then an *E. coli *production system may aid cost-effective production.

## Background

The APS is a multi-system autoimmune disease characterised by vascular thrombosis and/or recurrent pregnancy loss in patients who test positive for either aPL or lupus anticoagulant [1]. APS carries a significant burden of morbidity and mortality [2] with long-term anticoagulation being the only treatment with any proven benefit in reducing recurrent thrombosis [3]. Since anticoagulation carries an inherent risk of bleeding it is desirable to develop alternative treatments that target aPL directly. Patients with APS generally have high levels of serum IgG aPL. Monoclonal and polyclonal IgG aPL have been shown to be pathogenic and promote thrombosis *in vivo *[4,5]. aPL from patients with thrombosis only bind phospholipids (PL) in the presence of protein co-factors, of which β_2_GPI has been studied the most extensively. Understanding how these pathogenic aPL interact with β_2_GPI at the molecular level could ultimately facilitate the development of targeted therapies.

β_2_GPI contains five homologous domains [6] and anchors to PL via domain V [7]. Using domain deletion studies, in which β_2_GPI was produced with one or more domains deleted, it was shown that the amino terminal domain (DI) is particularly important for aPL binding, suggesting that crucial aPL binding epitopes are contained within this domain [8-10]. A compound based on DI is currently being studied for possible use as a toleragen to treat APS patients by inducing anergy in B lymphocytes that produce aPL [11]. Clearly an efficient method of DI production could enhance the scope for investigating the antigenic role of DI and facilitate epitope mapping studies. An expression system in *E. coli *could offer such a tool. Furthermore if DI is ultimately used therapeutically, prokaryotic expression lends itself to large-scale cost-efficient production.

*E. coli *is the most frequently used prokaryotic expression system for production of heterologous proteins due to its efficiency, cost-effectiveness and potential for high-level production [12,13]. However, various properties of different genes, their transcribed mRNAs and protein products may preclude efficient eukaryotic protein expression in bacteria. Current expression methods for DI use baculovirus containing a cloned cDNA sequence of the DI gene to infect *Spodoptera frigiperda *insect cells [14]. This method of expression is relatively expensive, laborious and less amenable to being scaled-up in comparison to prokaryotic expression systems. Other than the formation of two disulphide bonds, no other post-translational modifications such as glycosylation are necessary for biologically active DI expression. Hence an established *E. coli *expression system could hold several significant advantages over the currently available system of production.

There are no published reports of DI expression in *E. coli*. Previous attempts to achieve this may have been unsuccessful for a number of reasons. We hypothesise that these reasons are related to *E. coli *codon bias against eukaryotic codons, potential toxicity of DI to *E. coli *and the relative difficulty of disulphide bond formation in the reducing environment of bacterial cytoplasm. In this paper we have addressed these problems and now report the first system for expressing DI in *E. coli*. We have demonstrated the binding properties of the expressed protein to murine monoclonal anti-antibodies that recognise conformational epitopes on the surface of DI, to pathogenic human monoclonal IgG aPL and to polyclonal IgG derived from APS patients.

## Results and Discussion

### Pan gene codon optimisation and tight regulation of expression are critical in ensuring efficient production of DI by *E. coli*

**Figure 1:**
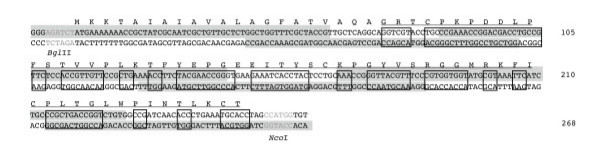
**Synthetic gene encoding for DI of human β_2_GPI**. The synthetic gene encodes for DI (underlined amino-acids) and an *Omp*A leader sequence. Codons were chosen for optimal expression of DI in *E. coli *by Juniper, altering 67% of the native sequence (boxed codons). Synthetic oligonucleotide primers are denoted by grey shading; oligonucleotides of the upper DNA sequence are read 5' to 3' (left to right) and the oligonucleotides of the lower DNA sequence are read 3' to 5' (left to right). Restriction sites are denoted in light grey font.

A synthetic gene encoding for DI was designed by the Juniper programme. Six 60 mer overlapping oligonucleotide primers covering the length of DI were specifically designed by Juniper to minimise non-specific annealing. These primers were used in one-step recursive PCR [15] to create a DI gene (figure 1). Importantly, Juniper incorporates codons used preferentially by *E. coli*, as the native cDNA sequence of DI contains an abundance of codons used rarely by *E. coli *[6]. Of the 61 codons present in the native DI gene, 26 (43%) are used by *E. coli *at a frequency of less than 1%. These rare codons are present in clusters of between two to five codons in length. Hence one may expect translational problems in *E. coli *with an abundant mRNA species containing an excess of rare low tRNA codons [16]. Even a single rare codon can have a deleterious effect on heterologous protein expression [17]. The establishment of other eukaryotic protein expression systems in *E. coli*, utilising the same codon optimisation principles, has been described elsewhere [17-19], but few, to our knowledge, have targeted all codons amenable to *E. coli *optimisation as we have done. It is planned for this software (Juniper) to be freely available over the internet in the near future and enquiries about this should be addressed to the authors of this paper. The proposed website is cited in the reference list.

**Figure 2:**
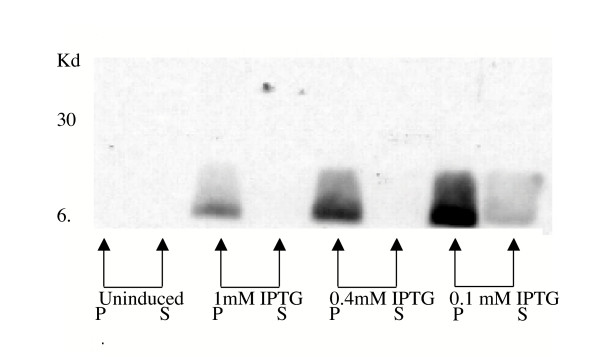
**Optimum DI expression observed at lower levels of IPTG induction**. Western blot of expression cultures induced with 1 mM, 0.4 mM, 0.1 mM IPTG and an uninduced control. Both non-reduced periplasmic extracts (P) and supernatant (S) samples were analysed using 15% SDS-PAGE and transferred onto a membrane. The membrane was probed with 6C4C10, a monoclonal murine anti-DI antibody. All cultures were performed in parallel under identical conditions and equal volumes applied to the protein gel. Greatest expression of DI was observed from cultures induced with 0.1 mM IPTG.

The eukaryotic protein DI is toxic to *E. coli*. Even small amounts of DI basal production resulted in cell death necessitating tight regulation of expression using the T7*lac *promoter [20] and the addition of 1% glucose to the expression cultures [21]. When the cell cultures were induced with 1 mM or 0.4 mM isopropylβ-D-thiogalactoside (IPTG), marked inhibition of cell growth occurred. However, inducing with 0.1 mM IPTG resulted in approximately 80% increase in OD_600 _values, suggesting that *E. coli *can only sustain a certain level of DI production, above which marked toxicity and cell death occurs. The incorporated *pel*B leader sequence directed the expressed target protein into the bacterial periplasmic compartment facilitating the formation of disulphide bond formation and hence correct folding of DI. Western blot analysis of periplasmic fractions from cell cultures induced with no IPTG, 0.1 mM IPTG, 0.4 mM IPTG and 1 mM IPTG indicate that most expression of target protein is seen with cell cultures induced with 0.1 mM IPTG, with some leakage of DI into the culture supernatant (figure 2). Furthermore, no target protein is seen in the uninduced control confirming the tight regulation of basal expression that this system provides. We suggest that *E. coli *may be able to sustain a critical level of DI production with gentle induction at low IPTG concentrations, above which intolerable amounts of rapid DI production result in marked *E. coli *toxicity, leading to overall reduced yield of target protein.

**Figure 3:**
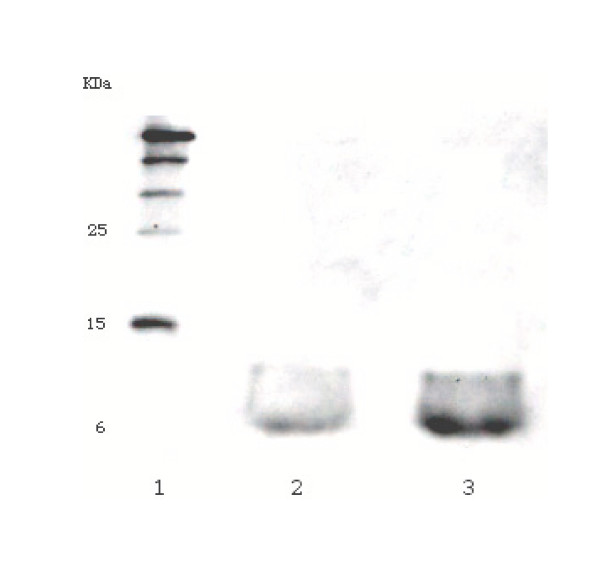
**Greater expression of DI seen with gene optimised for *E. coli *expression**. Western blot analyses of recombinant his_6_-tagged DI. Protein was transferred onto a nitrocellulose membrane from a 15%-SDS-PAGE gel and probed with an anti-his_5 _antibody. *Lane 1*, his_6_-tagged molecular weight protein markers; *lane 2*, DI expressed using the native cDNA human sequence; *lane 3*, DI expressed using sequence optimised for *E. coli*. Equal volumes of periplasmic extract were applied to lanes 1 and 2 on the protein gel.

In order to assess the contribution of using optimal codons preferred by *E. coli*, we compared expression of the synthetic bacterial gene with expression of human cDNA encoding DI. This human DI cDNA, cloned into a BacPAK plasmid, BD Biosciences (San Jose, CA, USA) was donated as kind gift from M Iverson and M Linnik (LJP, San Diego, USA). The human DI sequence was amplified using PCR with primers designed to incorporate flanking *Nco*I and *Xho*I restriction sites, which were used for ligation into pET-26b(+). Parallel expression cultures of both constructs (each identical other than one containing the native DI sequence and the other the synthetic DI sequence designed by Juniper) were performed. Equal amounts of periplasmic protein extracts from both cultures were transferred onto a membrane. Western blot analyses by probing with an anti-his_5 _antibody confirmed significantly greater target protein production using the synthetic gene (figure 3).

### Recombinant periplasmic his-tagged DI is conformationally correct and may be purified using nickel chromatography

**Figure 4:**
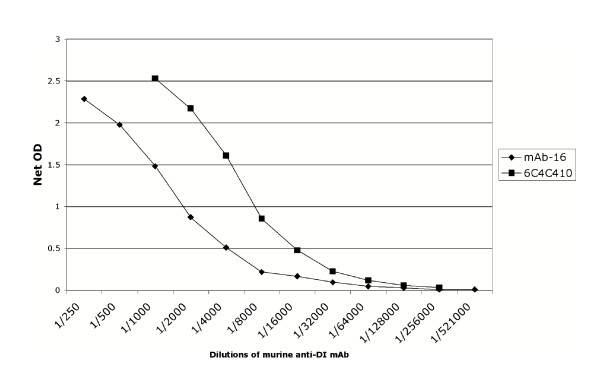
**Direct ELISA of murine-anti DI antibodies binding wild-type recombinant his_6_-tag DI**. 6C4C10 and mAb-16 bind conformational epitopes on DI. Binding of these antibodies to DI indicates that expressed recombinant DI is likely to be folded correctly.

The presence of hexahistidine (his_6_)-tagged DI within periplasmic samples was confirmed by performing Western blot experiments probing the membrane with mAb-16 and 6C4C10 murine anti-DI antibodies (figure 2) and an anti-his_5 _antibody (figure 3). The murine anti-DI antibodies, produced by immunizing mice with recombinant DI, recognize conformational epitopes of DI [22]. These antibodies have been shown previously to bind non-reduced DI on a Western blot but not to DI where the cysteine residues have been blocked to prevent disulphide bond formation. The fact that these antibodies bind expressed non-reduced DI on both Western blot (figure 2) and direct immunoassay (figure 4) suggests strongly that our periplasmic product is folded correctly.

**Figure 5:**
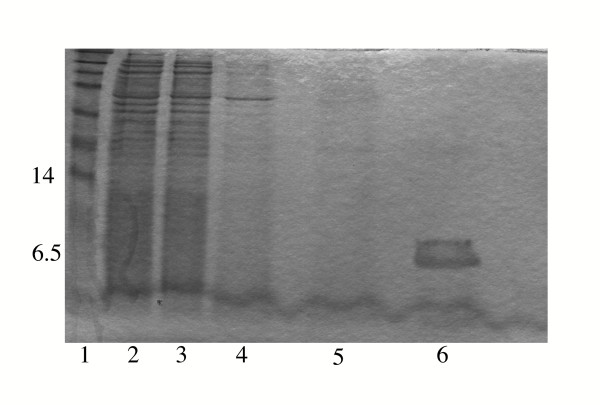
**DI purification using nickel chromatography**. Samples analysed by 15% SDS-PAGE. *Lane 1*, protein molecular weight marker; *lane 2*, periplasmic extract; *lane 3*, flow-through from nickel column; *lane 4*, wash with 5 mM imidazole; *lane 5*, wash with 45 mM imidazole; *lane 6*, elution with 300 mM imidazole.

DI was purified using nickel chromatography utilising the incorporated C-terminal his_6_-tag and eluted with 300 mM imidazole. Purity of the eluted DI protein was assessed using 15% SDS-PAGE after extensive dialysis against phosphate buffered saline (PBS)-10% glycerol, in order to remove the imidazole (figure 5). Yields of expressed soluble DI approximated to 750 μg/L expression culture, as assessed using the BCA protein assay.

### Human IgG aPL binds to expressed DI

**Figure 6:**
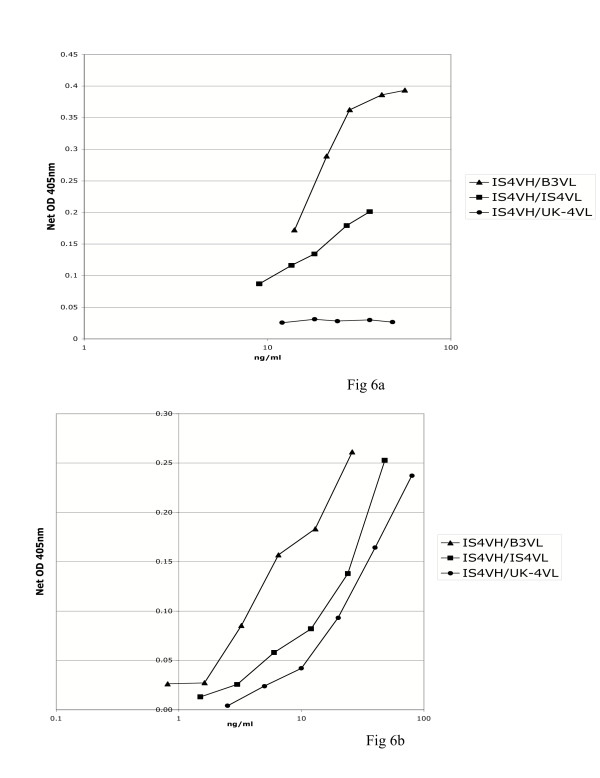
**Recombinant DI binds monoclonal human aPL with the same order of binding as seen with whole β_2_GPI**. ELISA of β_2_GPI (fig. 6a) and his_6_-tagged DI (fig. 6b) binding to IS4V_H_/B3V_L_. IS4V_H_/IS4V_L _and IS4V_H_/UK-4V_L_. The same order of binding of aPL to both β_2_GPI and DI was observed. Greatest binding was seen with IS4V_H_/B3V_L _and least with IS4V_H_/UK-4V_L_.

**Figure 7:**
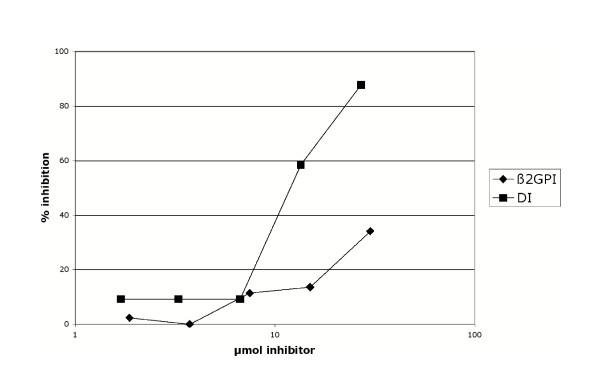
**Recombinant DI in solution is an effective inhibitor of a pathogenic monoclonal human aPL *in vitro***. Competitive inhibition of affinity purified IS4V_H_/IS4V_L _binding to his_6_tagged-DI bound to a nickel chelate plate, by recombinant DI and wild type β_2_GPI as inhibitors. A constant amount of antibody was mixed with varying concentrations of inhibitor and allowed to equilibrate at room temperature. For a given μmol concentration of inhibitor, DI alone is a more effective inhibitor of IS4V_H_/IS4V_L _than whole β_2_GPI.

IS4 is a monoclonal aPL with proven *in vivo *thrombogenic pathogenicity derived from a patient with APS [23]. Our group have made two variants of this monoclonal aPL, swapping the light chain (V_L_) with that of an anti-DNA antibody B3 and another monoclonal aPL UK-4. We have previously shown that the three expressed heavy/light chain combinations IS4V_H_/IS4V_L_, IS4V_H_/B3V_L _and IS4V_H_/UK-4V_L _possess different abilities to bind cardiolipin and β_2_GPI [24,25]. We have established that replacing the V_L _of IS4 with that of B3 (IS4V_H_/B3V_L_) increases binding to cardiolipin and β_2_GPI whereas IS4V_H_/UK-4V_L _binds very poorly to either antigen [24,25]. These three heavy/light combinations show similar order of binding to DI in direct enzyme-linked immunosorbent assay (ELISA) as was previously seen in anti-cardiolipin and anti-β_2_GPI ELISA (figure 6a and 6b) [24,25]. However, it was noted that though IS4V_H_/UK-4V_L _bound DI to a lesser degree than the other antibodies studied (figure 6b), the degree of binding to DI of this antibody was much greater than that observed to β_2_GPI (figure 6a) or cardiolipin [24,25]. These results suggest the presence of crucial antibody binding epitopes that become exposed on the surface of DI when it is not attached to the rest of the β_2_GPI molecule. This notion is underlined by the inhibition assay (figure 7), which demonstrates that expressed DI inhibits binding of affinity purified native IS4 to DI more strongly than equivalent μmol concentrations of whole β_2_GPI. This data confirms the antigenicity of recombinant DI expressed by *E. coli *in both solid and fluid phase assays.

**Figure 8:**
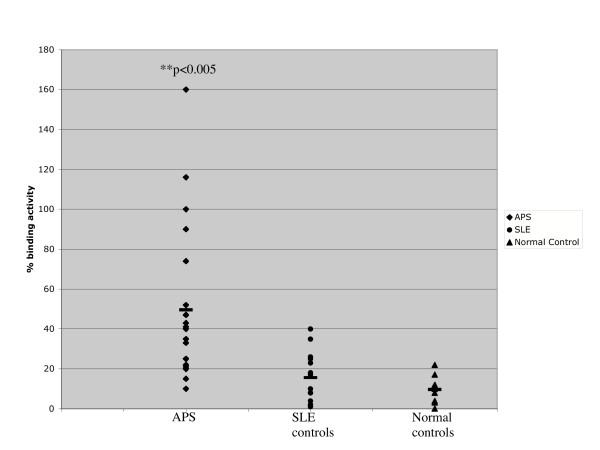
**Recombinant DI binds purified aPL derived from patients with APS**. Results are expressed as percentage binding of a standard APS IgG sample known to bind DI, whole β_2_GPI and CL. IgG was purified from 21 APS patients, 14 SLE patients and 9 normal controls. 20 μg/ml of IgG of each individual patient sample was tested for binding to DI and compared to the standard. Binding to DI was significantly greater in the APS group compared to SLE disease control and normal control groups (Student's t-test analysis (paired, 2-tailed) – APS versus SLE, **p < 0.005; APS versus normal controls, **p < 0.005; SLE versus normal controls p = 0.2).

Antigenicity studies of recombinant DI produced by *E. coli *were expanded to test binding to a series of affinity purified polyclonal IgG samples derived from patients with APS. 21 serum samples known to bind cardiolipin and β_2_GPI from patients with APS were identified. The IgG fraction was affinity purified using Protein G coated beads and tested for binding to recombinant DI coated on a nickel plate. We also tested purified IgG derived from two sets of control subjects. There were nine healthy controls and 14 subjects with systemic lupus erythematosus (SLE) who did not have APS. SLE was chosen as a disease control because it is a closely related autoimmune disease and SLE and APS can co-exist in the same patient. Figure 8 shows that binding of polyclonal IgG from patients with APS was significantly greater than binding to polyclonal IgG from either control group.

Clearly recombinant DI expressed by *E. coli *binds IgG from patients with APS. The fact that DI binds IS4 better than whole β_2_GPI in the fluid phase implies that recombinant DI may be used as a competitive inhibitor of the pathogenic aPL/β_2_GPI interaction and thus act as a potential therapeutic agent. The ability to produce such an agent by expression in *E. coli *would be advantageous. Given that DI is produced in bacteria however sufficient measures should be undertaken to ensure expressed samples are free from lipopolysaccharide.

## Conclusion

We have shown that, by using a stepwise strategy to address specific problems relating to codon optimisation, periplasmic protein translocation and tight regulation of expression, efficient bacterial expression of DI of human β_2_GPI is possible. Recombinant DI was conformationally correct and bound human monoclonal aPL in both solid and fluid phase assays. DI also bound a series of IgG samples derived from patients with APS. This is the first description of DI production using an *E. coli *expression system. Ease and efficiency of expression will be utilised to study different epitopes on DI and investigate the binding of variants of DI to polyclonal IgG affinity purified aPL derived from patients with APS. Furthermore if DI or a peptide derived from DI is eventually used in the therapeutic setting [11] an *E. coli *system of production would be likely to facilitate production and cost.

Finally, we submit that the simple design and production of a synthetic gene globally optimised for expression in *E. coli *using Juniper and one-step recursive PCR, as illustrated in this paper, is an important technique that can be applied to other eukaryotic proteins, particularly if the cDNA sequences of these genes have clusters of codons used infrequently by *E. coli*.

## Methods

### Materials

Oligonucleotide primers were synthesised by Thermobiosciences (Germany) and used without further purification. Restriction enzymes *Nco*I, *Xho*I and *Bgl*II and T4 DNA ligase were purchased from Promega, (Southampton, UK). Plasmids pET-26b(+) and BL21(DE3) *E. coli *cells were purchased from Novagen (Nottingham, UK). DH5α *E. coli *cells were supplied by Gibco (Paisley, UK). Automated sequencing was carried out by MWG-Biotech (Ebensburg, Germany).

### Monoclonal antibodies

Three human monoclonal antibodies (mAb) were produced in Chinese Hamster Ovary (CHO) cells, an *in-vitro *expression system, which has been described in detail in previous papers [25,26]. Affinity purification of the antibodies from these cells was carried out by Chemicon Europe Ltd, Southampton, UK. Murine monoclonal anti-human DI antibodies mAb-16 and 6C4C10 were kind gifts from Dr M Linnik and Dr M Iverson, La Jolla Pharmaceuticals (LJP), California, USA.

### Production of a construct containing a synthetic gene encoding the DI sequence

A synthetic gene was designed to encode for DI of human β_2_GPI, an N-terminal *Omp*A leader sequence and *Bgl*II/*Nco*I flanking restriction sites. This gene was designed by the computer programme Juniper, which designed six 60 mer overlapping oligonucleotide primers based on the published amino acid sequence of DI [6]. The gene encoding for DI was synthesised using these primers by recursive PCR [15] (figure 1). Twenty pmol of each outer primer and two pmol of each internal primer were used in a reaction containing 2 U of *Vent *DNA polymerase (New England Biolabs (NEB), Hertfordshire, UK) and 25 mM of 2'-deoxynucleoside 5'-triphosphates (dNTPs') in 100 μl of the 10× supplied buffer and ddH_2_0. PCR was performed under the following conditions: 95°C for 8 min, 30 cycles of 94°C for 2 min, 57°C for 1 min, 72°C for 1 min with a final extension step of 72°C for 10 min. This gene was then ligated into the expression plasmid and sequenced to exclude PCR errors.

The pET system originally described by Studier and Moffat [27] was used to express recombinant DI. A variety of constructs were used to optimise expression. The final cloned expression construct encoded for a *pel*B leader sequence followed by DI and a C-terminal his_6_-tag cloned into pET-26b(+). Both the leader and the his_6_-tag are present within the pET-26b(+) plasmid so that the *Omp*A leader sequence that had originally been created by recursive PCR (figure 1) was redundant and removed at this stage. The target gene was under the control of the high stringency phage T7*lac *promoter [20] and a strong T7 translation initiation site.

### Induction experiments

The final recombinant expression vector pET26b(+), containing the DI gene and a kanamycin resistance gene, was transferred to the expression strain BL21(DE3). Single colonies were picked from the transformants, and 5-ml cultures prepared in 50-ml falcons (overnight with shaking, 30°C). Fresh 500-ml cultures in 2.5-litre flasks were then set up using the overnight culture to inoculate the medium to an optical density (OD)_600 _of 0.1. Cultures were induced with a range of IPTG (0.1 mM, 0.4 mM or 1 mM) at an OD_600 _of 0.6 and allowed to grow for a further 4 hours (30°C, shaking). 'Terrific' broth containing 60 μg/ml of kanamycin was used to prepare the overnight cultures with the addition of 1% glucose for the expression cultures. OD_600 _was recorded at periodic intervals before and after induction as a measure of bacterial growth in the culture.

### Preparation of periplasmic fraction and purification

The method for periplasmic extraction of protein from *E. coli *is based on previously published methods by our group for the expression of periplasmic Fabs of human autoantibodies in W3110 *E. coli *[28]. Briefly, cultures were spun (4000 g, 20 min, 4°C) following 4 hours induction and supernatant was saved (at 4°C) for the detection of any leaked DI protein from the cells. The cell pellet was then exposed to osmotic shock by suspension in ice-cold water (30 ml of dH_2_0 per litre of culture), stirred for 30 min at 4°C, and spun (8000 g, 20 min, 4°C). This supernatant constitutes the periplasmic fraction containing recombinant DI, an aliquot of which was stored at -20°C for subsequent SDS-PAGE and Western blot analysis.

For purification periplasmic extract was loaded on a column containing nickel charged resin, Novagen (Nottingham, UK). DI bound to the column by virtue of the C-terminal his_6_-tag and was eluted with 300 mM imidazole, (2 M NaCl, 80 mM Tris-HCl, pH 7.9). Recombinant his_6_-tagged DI was then dialysed extensively against PBS-10% glycerol overnight at 4°C using dialysis visking tubing, MWCO-3500, Medicell (London, UK). The purity of the eluted DI was assessed on SDS-PAGE 15% gels.

### Functional assessment

#### Affinity purification of IgG aPL

Polyclonal IgG was purified from 21 patients satisfying the preliminary diagnostic criteria for APS [1]. IgG was also purified from 9 normal controls and 14 patients with SLE (but no APS) as disease controls. 19/21 patients with APS, 13/14 patients with SLE and 7/10 healthy controls were female. The mean ages of the subjects in the three groups were comparable (APS – 44.0 years, SD 7.0; SLE 37.2 years, SD 10.0; healthy controls 33.2 years, SD 9.0). Ethical approval for the study was granted by University College London Hospital Research Ethics Committee. Protein G beads (Amersham, Bucks, UK) were prepared by washing with PBS. 1 ml serum was mixed with 2 mls 0.02 M sodium phosphate (pH 7) binding buffer and incubated at room temperature (RT) for two hours with 0.5 ml prepared Protein G beads. This mixture was then spun (200 g, 5 min, 4°C) and the beads washed a further 3 times with binding buffer. Elution of IgG from the beads was performed by mixing the beads with 2 mls 0.1 M glycine (pH 2.7) for 1 min. This was spun (200 g, 5 min, 4°C) and the supernatant stored as the IgG fraction at -20°C. The amount of IgG was quantified using a direct IgG ELISA described in a previous paper [25].

#### Direct binding ELISA of aPL binding to purified recombinant his_6_-tagged DI

Nickel chelate-coated microwell plates, VH Bio (Gateshead, UK) were coated with 50 μl of recombinant purified his_6_-tagged DI and diluted to a concentration of 50 μg/ml using PBS. One half of the plate (the test wells) was coated with DI and the other half with PBS (the control wells). Plates were incubated at RT for 2 hours and were then washed three times with PBS, blocked with 100 μl of 0.25% gelatin, Sigma (Poole, UK) in PBS and incubated for a further 1 hour at RT. After washing the plates three times with PBS, 50 μl of a monoclonal human IgG aPL (IS4V_H_/IS4V_L_, IS4V_H_/B3V_L _or IS4V_H_/UK-4V_L_) derived from CHO cell culture supernatant [24] in sample, enzyme and conjugate dilution (SEC) buffer (100 mM Tris-HCl (pH 7), 100 mM NaCl, 0.02% Tween-20 and 0.2% BSA) was added at concentrations ranging from 9 ng/ml to 80 ng/ml. Serial dilutions of mAb were loaded such that for each dilution loaded in a test well, there was a corresponding control well loaded with the same dilution. For polyclonal IgG aPL purified from APS patients, SLE and normal controls, 50 μl of 20 μg/ml of IgG in SEC buffer was added to each well. Binding of human antibodies to the plate was detected by adding goat anti-human IgG alkaline phosphatase conjugate, Sigma (Poole, UK) diluted 1:2000 in SEC. After incubation at 37°C for 1 hour, bound antibody was detected by addition of alkaline phosphatase chromogenic substrate. The OD was measured in a Genios microplate autoreader (Tecan, Reading, UK). A net OD was calculated for each well to take into account background (OD test well – OD control well). Results of polyclonal IgG were expressed as a percentage binding of a standard APS patient sample known to bind DI, whole β_2_GPI and CL.

#### Competitive inhibition ELISA

A direct binding ELISA carried out as above was used to determine the concentration of native affinity purified antibody IS4V_H_/IS4V_L _required to achieve ~50% maximum binding. This concentration was 200 ng/ml. DI and β_2_GPI as test inhibitors were diluted in PBS at concentrations ranging from 0 (i.e. no inhibitor) to 30 μM. Affinity-purified IS4V_H_/IS4V_L _was then added to each concentration of inhibitor to achieve 200 ng/ml final concentration of antibody for each sample. The samples were incubated at RT for 2 hours and then tested for binding to DI on an ELISA plate as described earlier for the direct ELISA above. The per cent inhibition for each concentration of inhibitor was determined from the following formula:

% inhibition=(A_0_-A/A_0_) × 100, where A is the OD from the well containing the inhibitor (corrected for background) and A_0 _is the OD from the well containing no inhibitor (corrected for background).

## Abbreviations

β_2_GPI, beta 2 glycoprotein I; aPL, antiphospholipid antibodies; APS, antiphospholipid syndrome; DI, domain I; *E. coli*, *Escherichia coli*; PL, phospholipid; mAb, monoclonal antibodies; V_H_, heavy chain; V_L_, light chain; IPTG, isopropylβ-D-thiogalactoside; OD, optical density; SLE, systemic lupus erythematosus.

## Authors' contributions

YI performed the recursive PCR and cloning work, optimised and established the expression system and performed the immunoassays. IG and AL performed CHO expression of human monoclonal aPL used in the immunoassays. CR and LP developed Juniper and the technique of recursive PCR and gave relevant technical assistance. DSL, DAI and AR were involved in concept development and experimental design. All authors have seen and approved this work.
